# Kartagener’s Syndrome: A Case Series

**DOI:** 10.7759/cureus.61722

**Published:** 2024-06-05

**Authors:** Nisrine El Marzouki, Fatima Zahra Alaoui-Inboui, Bouchra Slaoui

**Affiliations:** 1 Pediatric Pneumo-Allergology Unit, Pediatric Department 2, Hôpital Mère-Enfant Abderrahim Harouchi, Centre Hospitalier Universitaire Ibn Rochd, Casablanca, MAR

**Keywords:** dextrocardia, situs inversus, sinusitis, bronchiectasis, kartagener's syndrome

## Abstract

Kartagener’s syndrome is an uncommon autosomal recessive ciliary dyskinesia. It combines a triad comprised of bronchiectasis, chronic sinusitis, and situs inversus. This work aims to describe the clinical and paraclinical aspects of primary ciliary dyskinesia using Kartagener's syndrome as a model and to highlight the difficulties of confirming the diagnosis in our context. We report four observations (three boys and one girl with an average age of 10 years) of Kartagener's syndrome collected in the department of pediatric pneumo-allergology. Chronic bronchorrhea and otorhinolaryngological manifestations were found in all cases. Signs of neonatal respiratory distress syndrome were found in only one case. One child had dysmorphic facial features suggestive of Noonan's syndrome and conductive hearing loss. Digital hippocratism was found in half of the cases, along with pulmonary crackles and heart sounds perceived on the right. A chest CT scan showed bronchiectasis in all patients and necrotic adenopathy suggestive of tuberculosis in one case. Sinus imaging showed an appearance of pansinusitis. All children had abdominal situs inversus with dextrocardia. They had received antibiotic therapy with amoxicillin-clavulanic acid associated with respiratory physiotherapy. The girl had benefited from a right lobectomy with a follow-up of 18 months and a good evolution. In light of these four observations, Kartagener's syndrome is a rare disease but can be compatible with normal life if the treatment is done early. However, in our context, the difficulty of confirming the diagnosis explains its delay with the risk of progression of pulmonary lesions.

## Introduction

Primary ciliary dyskinesia (PCD) is a rare congenital disorder associated with a permanent and ubiquitous abnormality of ciliary structure and/or function. This rare autosomal recessive disease has a prevalence of 1/10,000-20,000 births [1]. In its respiratory form, cilia dysfunction in the upper and lower respiratory mucosa leads to early-onset chronic obstructive pulmonary disease, secondary to mucociliary clearance disorders and recurrent respiratory infections. Diagnosis is often difficult to confirm. Symptoms are not very specific, investigations are complex, and the diagnostic approach is not yet consensual. Diagnosis is usually confirmed several years after the onset of symptoms or even in adulthood [2]. PCD is manifested by lateralization of the organs in 50% of cases, resulting in Kartagener's syndrome, which combines bronchiectasis, situs inversus, and chronic sinusitis [3]. Early diagnosis is crucial for preserving lung function, quality of life, and life expectancy and preventing complications. This work aims to describe the clinical and paraclinical aspects of PCD, taking Kartagener's syndrome as a model, and to highlight the difficulties of confirming the diagnosis in our context.

## Case presentation

Case 1

We report the case of a 16-year-old teenager from a first-degree consanguineous union who had consulted us for recurrent respiratory infections. He was being followed for hearing loss and nasal polyposis under medical treatment. This child had been suffering from a chronic daytime and nocturnal cough since infancy, with expectoration during the daytime for one year. This was complicated by the onset of dyspnea on exertion, associated with left basi-thoracic pain and exaggerated bronchorrhea that had become greenish.

**Figure 1:**
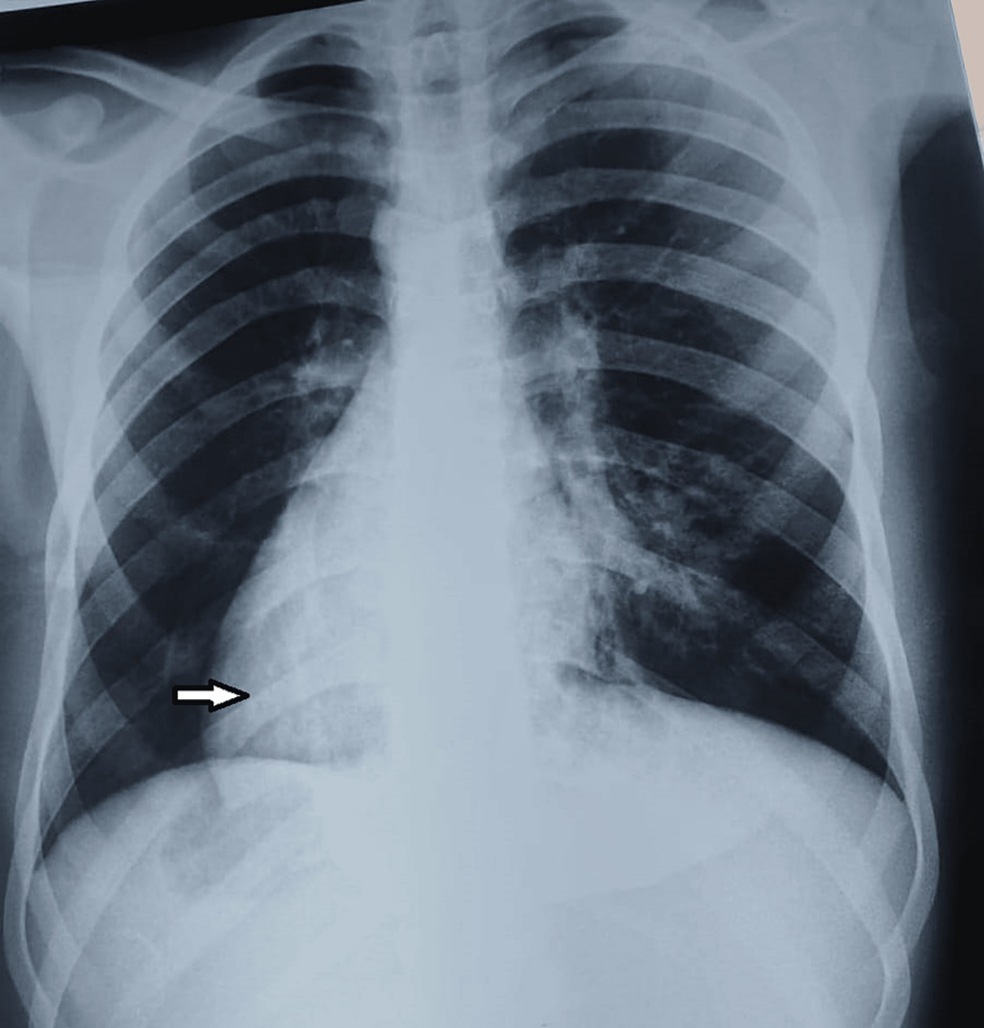
Chest X-ray showing the situs inversus (white arrow), bronchial syndrome, and alveolar focus of the lingula

On clinical examination, the child was apyretic and eupneic with a respiratory rate of 18 cycles per minute and left basi-thoracic crackles. Heart sounds were heard on the right. Oxygen saturation (SpO2) was 94%. The otolaryngological examination revealed hearing loss and translucent nasal polyposis, more marked on the right. The chest X-ray showed a right heart tip and bilateral bronchial syndrome with the ascension of the left diaphragmatic dome (Figure 1).

**Figure 2:**
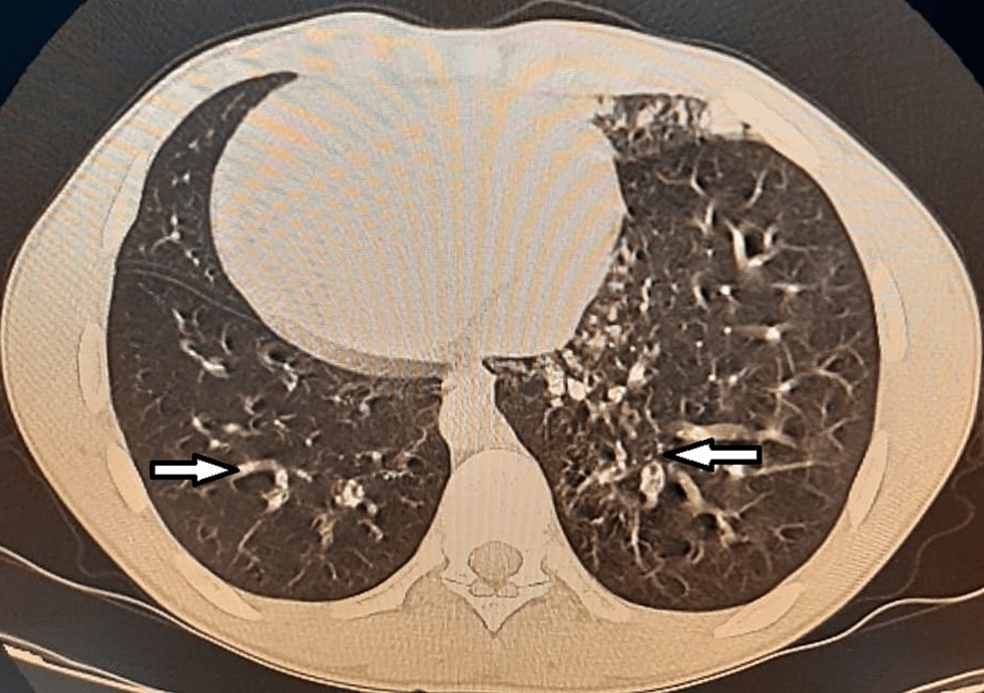
Chest CT scan (parenchymal window) showing the bilateral bronchial dilatation (white arrows) associated with situs inversus

The thoracic scanner revealed superinfected bilateral bronchial dilatations associated with situs inversus (Figure 2).

**Figure 3:**
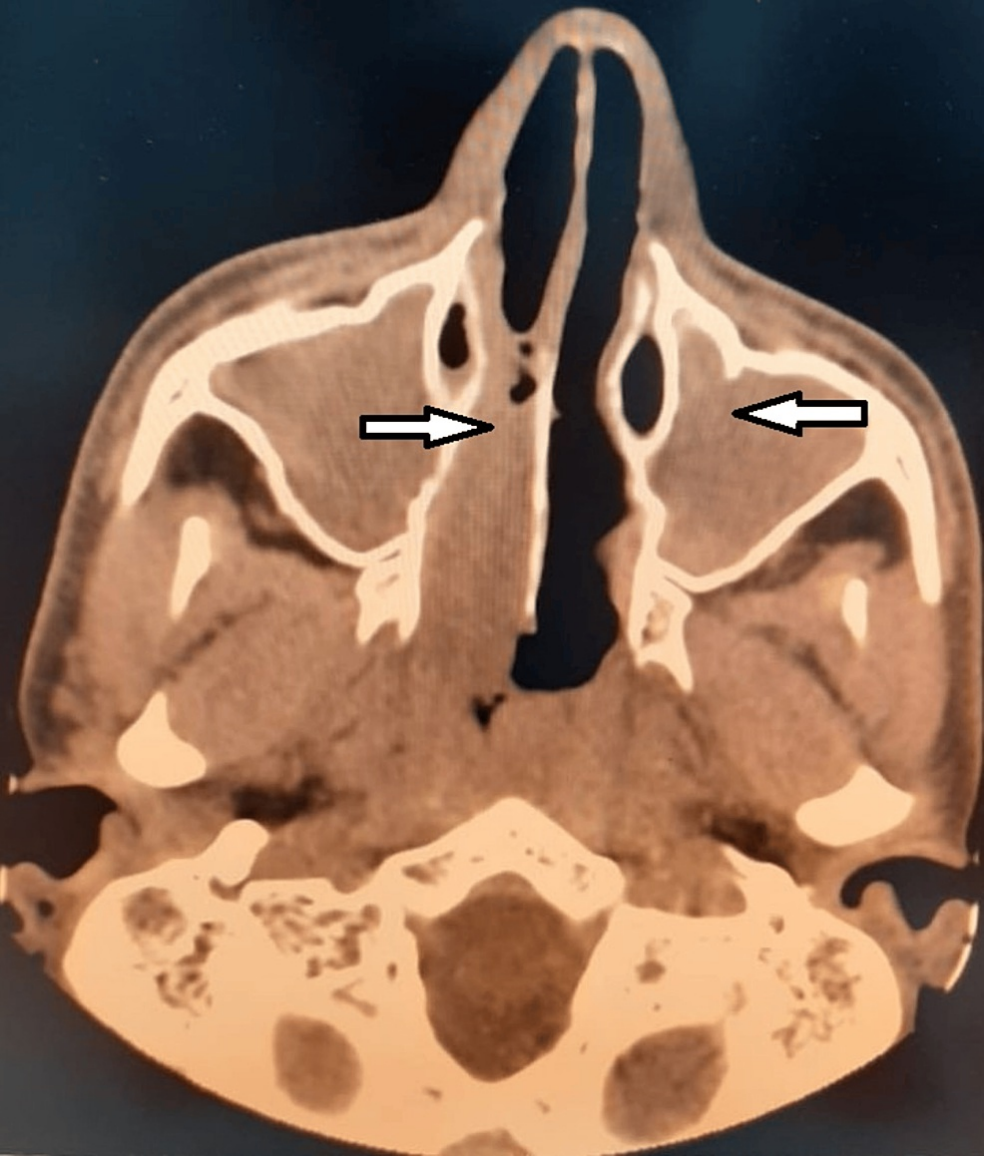
Sinus CT scan showing chronic sinusitis with nasosinusal polyposis (white arrows)

A sinus CT scan showed chronic sinusitis with nasosinus polyposis (Figure 3).

Cytobacteriological examination of the sputum revealed gram-positive cocci on direct examination, with a negative culture. Bronchial aspiration during bronchoscopy revealed no Koch's bacillus on direct examination, with a negative mycological examination. The HIV serology was negative, and the immunoglobulin assay was normal. Bronchoscopic examination showed no tumors, granulomas, or visible foreign bodies.

To rule out amyloidosis, urea, creatinine, and 24-hour proteinuria were measured, all within the normal range, and a labial biopsy showed chronic sialadenitis, Chisholm and Masson grade I. Respiratory function tests did not reveal any obstructive or restrictive syndromes. Blood gas levels were normal. Given this clinical and laboratory picture, the diagnosis was superinfected bronchial dilatation, revealing Kartagener's syndrome. The patient was treated with amoxicillin-clavulanic acid-based antibiotics, combined with respiratory physiotherapy.

Case 2

A 12-year-old boy from a non-consanguineous marriage was hospitalized for febrile respiratory distress. He had a history of hospitalization for neonatal respiratory distress. For the past two years, he has been suffering from a chronic diurnal and nocturnal cough, especially morning sputum, for three months. He later presented with febrile dyspnea associated with greenish sputum. On clinical examination, the child was febrile at 38.5°C, pale, and polypneic at 36 cycles per minute, with signs of respiratory distress such as intercostal and subcostal retractions and bilateral grunting on pulmonary auscultation. The sounds of the heart were heard on the right. The SpO2 was 94%. He reported failure to thrive at -2 standard deviations.

A chest X-ray showed a right heart silhouette, bilateral bronchial syndrome, left basal opacity, and encysted pleural effusion. A chest CT showed situs inversus, bronchiectasis of the lingula, bilateral pneumopathy with parenchymal micronodules, and necrotic subcarinal adenopathy. A CT scan of the sinuses showed chronic pansinusitis. Abdominal ultrasounds showed abdominal situs inversus. Cardiac ultrasound showed dextrocardia with no other abnormalities. Cytobacteriological examinations of the sputum were sterile. The three BK sputum tests revealed the presence of acid-fast bacilli.

The sweat test was negative. HIV serology was negative. Plasma protein electrophoresis showed a moderate inflammatory syndrome. Immunoglobulin and lymphocyte subpopulation assays were normal. The diagnosis was pulmonary tuberculosis grafted onto Kartagener's syndrome.

The child was started on amoxicillin-clavulanic acid and two months of rifampicin, isoniazid, pyrazinamide, and ethambutol in the attack phase, then four months of rifampicin and isoniazid as an antibacillary treatment protocol, combined with respiratory physiotherapy.

Case 3

A seven-year-old girl from a first-degree consanguineous marriage was hospitalized for respiratory distress. This child had been suffering from a chronic cough for three years with sputum, especially in the morning. On clinical examination, the child was pale, with failure to thrive at -2 standard deviations, digital hippocratism, respiratory rate of 31 cycles per minute, diffuse rales on pulmonary auscultation, heart sounds perceived on the right, and SpO2 of 98%. An ENT examination showed no acute otitis media or seromucous otitis.

A chest X-ray showed a cardiac silhouette on the right. Thoracoabdominal CT revealed a complete situs inversus in the thoracic and abdominal regions and multiple foci of cystic and cylindrical bronchial dilatation in the dorsal segment of the right upper lobe, the lingula, the middle lobe, and predominantly in the right lower lobe. Cardiac ultrasound showed dextrocardia with no other abnormalities. Sinus radiography showed an appearance consistent with pansinusitis.

Based on these clinical and imaging findings, the diagnosis of Kartagener's syndrome was made. Cytobacteriological examinations of the sputum revealed gram-positive cocci, and the culture was sterile. The child was started on amoxicillin-clavulanic acid antibiotics combined with respiratory physiotherapy. The child underwent a right lower lobectomy. During the 18-month post-procedure follow-up, we noted a regression of cough and morning sputum.

Case 4

A five-year-old, four-month-old child from a first-degree consanguineous marriage was hospitalized for febrile respiratory distress. He had been suffering from recurrent respiratory infections since the age of six months and had been pronounced deaf since the age of two years. On clinical examination, the child was febrile at 39°C, had intense subcostal and intercostal retractions, a respiratory rate of 60 cycles per minute, and bilateral crackles and wheezing on pulmonary auscultation.

**Figure 4:**
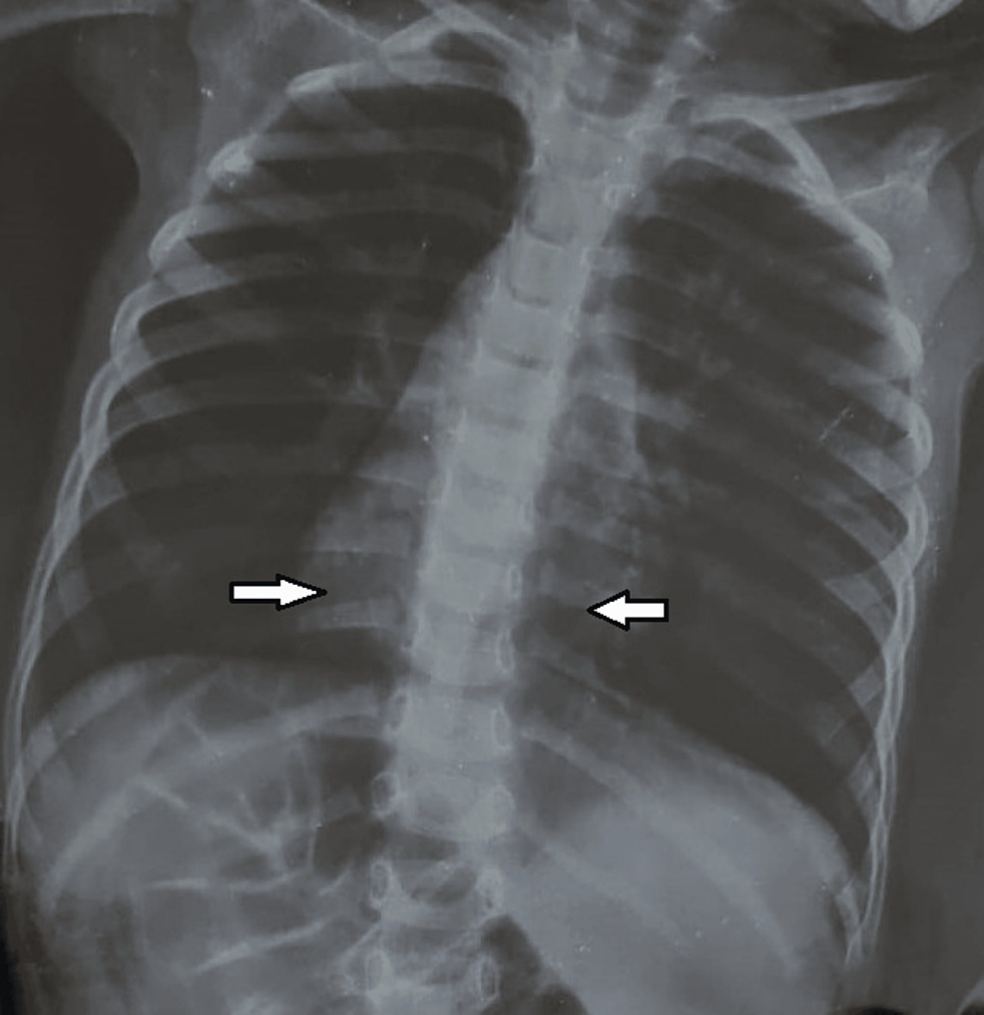
Chest X-ray showing dextrocardia (right arrow) and left upper lobar pneumonia with minimal homolateral pleural effusion (left arrow)

Heart sounds were heard on the right, and SpO2 was 92%. His facial features were suggestive of Noonan's syndrome, with a broad forehead, low-set ears, and a short neck with pterygium coli. His height and weight were behind -3 standard deviations. ENT examination revealed conductive hearing loss with bilateral profound hearing loss on auditory evoked potentials. A chest X-ray showed left upper lobar pneumonia with minimal homolateral pleural effusion (Figure 4).

**Figure 5:**
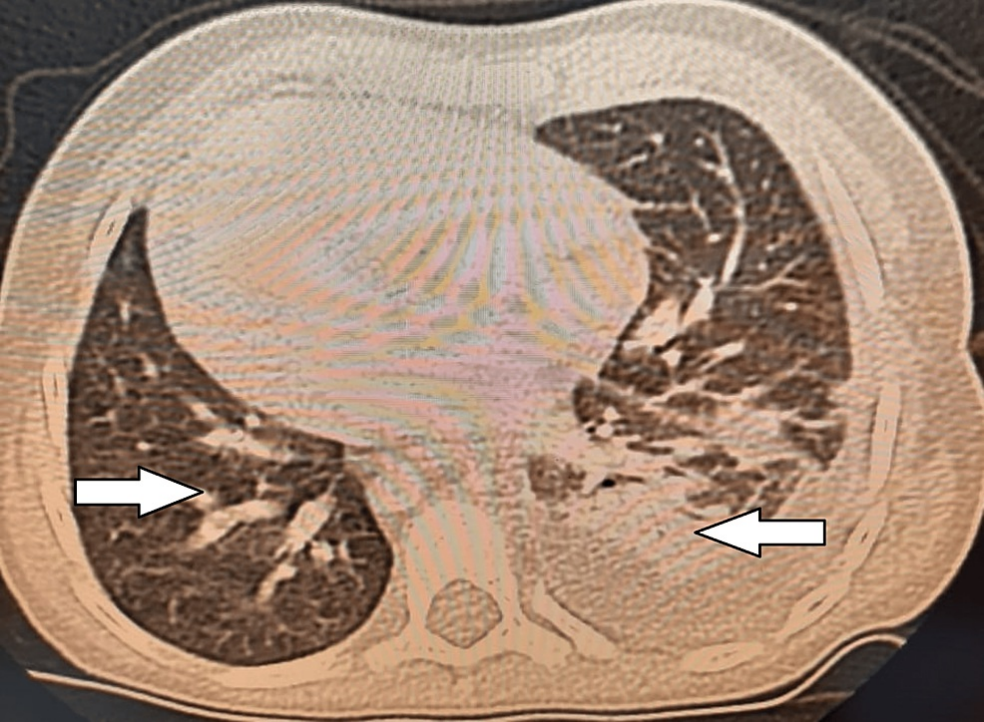
Thoracic CT scan (parenchymal window) showing situs inversus, bronchial dilatation (right arrow), and left laterobasal and posterobasal thoracic empyema (left arrow)

A chest CT showed a likely infectious left pleuro-pneumopathy with thoracic empyema and bronchial dilatation (Figure 5).

Cytobacteriological examinations isolated only coagulase-negative *Staphylococcus* on three occasions. Cardiac ultrasound showed dextrocardia with no other abnormalities. Abdominal ultrasound revealed a situs inversus with crossed renal ectopy and normal-looking kidneys. As part of the malformation work-up, cerebral and cervical MRIs were performed, revealing no abnormalities. In view of these findings, we concluded that Kartagener's syndrome is associated with a suspicion of Noonan's syndrome. The child was treated with antibiotics and respiratory physiotherapy. The evolution was marked by apyrexia and an improvement in respiratory status.

## Discussion

PCD, also known as immobile cilia syndrome, is a genetically determined disease caused by a hereditary dysfunction of the ciliary apparatus [4]. PCD is an autosomal recessive, heterogeneous group of disorders of ciliary ultrastructure and function. These are rare respiratory diseases, and their frequency is estimated at 1/10000 to 1/40000 [5]. The prevalence of this pathology is probably higher in our country, Morocco, which has a high rate of inbreeding. It is a disease responsible for a mucociliary purification defect leading to chronic and/or recurrent infections of the upper and lower airways beginning in childhood [2].

Kartagener's syndrome is a subtype of PCD characterized by a situs inversus accompanying the typical symptoms of PCD. Abnormalities in the primary cilia of embryonic nodal cells lead to abnormal nodal flow and visceral lateralization. A partial lateralization defect (heterotaxy) is found in 12% of PCD cases, such as simple dextrocardia, median liver, isomerism, or Ivemark syndrome [6]. Symptoms often begin in infancy or even the neonatal period, with chronic, non-specific respiratory symptoms, so it is generally not diagnosed [7].

The age of diagnosis varies from author to author and is often late. Some authors have reported cases in the neonatal period [8]. Indeed, the occurrence of unexplained neonatal respiratory distress in a newborn at term is very frequent, with an incidence varying from 44% to 85%. Respiratory symptoms are non-specific in newborns, ranging from transient tachypnea to prolonged oxygen dependence. These symptoms may be associated with radiological abnormalities such as segmental atelectasis. The anamnesis reveals parental consanguinity in around 20% of cases [6].

Respiratory signs are present in all patients. The association of a daily chronic hacking cough with early-onset ENT symptoms is highly suggestive of PCD. In small children, the main symptoms are a chronic cough associated with chronic bronchorrhea, complicated by exacerbations and/or acute bronchopulmonary infections [9]. Auscultation usually shows grunting, crackles, and sometimes wheezing sounds. The main extra-respiratory manifestation is visceral lateralization defects (mainly situs inversus totalis, but sometimes simple dextrocardia, median liver, polyspenia, isomerism, Ivemark syndrome, etc.), reported in 40-50% of patients [2,10,11]. Other rare malformations are associated with DCP, mainly cardiovascular, in 3-8% of patients [11]. These clinical elements are more associated than specific, and the elimination of certain differential diagnoses calls for further investigation of DCP [1].

The presence of bronchial dilatations predominating at the base in a child or adolescent should raise the possibility of PCD [12]. These bronchiectases are identified on a thoracic CT scan, which can be used to determine their anatomical type, extension, and even complications. Diagnosis is made when the intra-bronchial diameter is greater than that of the associated artery. They most often concern the middle and lower lobes [6,12].

Microbiological data from patients with PCD have been collected in several studies, varying considerably in group size [13,14]. The pathogens most frequently isolated from respiratory samples of children with PCD are *Haemophilus influenzae*, *Streptococcus pneumoniae*, and *Staphylococcus aureus* [15-17].

Positive non-tuberculous mycobacteria cultures can be found in up to 10% of PCD patients [15,17,18]. In cross-sectional studies, *Pseudomonas aeruginosa* has been isolated in around 10% of PCD patients [15-18]. There is no standard diagnosis for PCD [19], which is often difficult to confirm, despite the range of sophisticated diagnostic tests available. Diagnosis is based on clinical presentation, measurement of nasal nitric oxide, ciliary motile function analysis by high-speed video microscopy, transmission electron microscopy, and genotyping. Immunofluorescence remains a future prospect [20].

American recommendations suggest an early genetic analysis in the diagnostic approach (biallelic pathogenic variants in PCD-associated genes) [21]. In the populations studied, genetic defects have been identified in around 60% of cases, and many genes have yet to be identified [22].

Finally, gene mutations in PCD patients with normal ciliary ultrastructure offer irrefutable confirmation that PCD can occur in the absence of ciliary abnormalities on electron microscopy [10]. These explorations are not currently available in our context.

The guidelines of the European Respiratory Society and the American Thoracic Society insist that the demonstration of a pathogenic biallelic mutation or an X-linked hemizygous mutation in a known gene involved in PCD confirms the diagnosis [23].

At present, there is no specific etiological treatment. Treatment is essentially based on physiotherapy for bronchial drainage, combined with antibiotic therapy during periods of respiratory infections, vaccination (*Pneumococcus* and *Haemophilus influenzae*), and cardiac monitoring [24]. European guidelines recommend that children be seen once every three months for a respiratory assessment using a spirometer and sputum microbiology, with access to bronchoscopy and more specialized tests if necessary [25].

Antibiotic therapy is recommended in the event of clinical exacerbations or deterioration in pulmonary function testing. Antibiotic therapy is prolonged (≥10-15 days) and adapted to the latest microbiological examinations, with preference given to a broad spectrum (classically amoxicillin-clavulanic acid, cephalosporins, or cotrimoxazole) [2].

Lobectomy is not systematically proposed as a treatment for PCD. The decision to perform lobectomy in PCD requires multidisciplinary discussion between pulmonologists, anesthesiologists, and surgeons.

Although lobectomy may be beneficial in rare cases of PCD with severe, localized bronchiectasis, it should be considered with caution. In patients with end-stage respiratory failure, lung or heart-lung transplantation may be suggested [26].

ENT management is based on antibiotic therapy during exacerbations, combined with a background treatment adapted to the sinus and/or otological pathology. Nasal irrigation with isotonic serum is the mainstay of treatment for chronic rhinosinusitis [27]. Local corticosteroids may be useful in reducing mucosal inflammation, particularly in the presence of polyps, although they have not been studied in the context of PCD [28,29]. In the event of a hearing deficit affecting communication and acquisition, a hearing aid is proposed temporarily [2].

Kartagener's syndrome remains a rare, disabling disease, but one that can be compatible with a normal life if treated early. However, in forms with significant pulmonary lesions, the patient's prognosis is at risk in the short term due to severe multi-visceral damage [8].

## Conclusions

In light of these four observations, we reiterate that Kartagener's syndrome remains a rare genetic disease whose early, appropriate, and multidisciplinary management improves its evolutionary profile by delaying and decreasing the frequency of complications.
